# OTB-YOLO: An Enhanced Lightweight YOLO Architecture for UAV-Based Maize Tassel Detection

**DOI:** 10.3390/plants14172701

**Published:** 2025-08-29

**Authors:** Yu Han, Xingya Wang, Luyan Niu, Song Shi, Yingbo Gao, Kuijie Gong, Xia Zhang, Jiye Zheng

**Affiliations:** 1 Institute of Agricultural Information and Economics, Shandong Academy of Agricultural Sciences, Jinan 250100, China; 19726358251@163.com (Y.H.); nly83412@126.com (L.N.); 2 School of Physics Science and Information Engineering, Liaocheng University, Liaocheng 252000, China; wenerzhang2002@163.com; 3 Crop Research Institute, Shandong Academy of Agricultural Sciences, Jinan 250100, China; wangyaya2013@163.com (X.W.); gongkj@sina.com (K.G.); 4 Shandong Academy of Agricultural Machinery Sciences, Jinan 250100, China; shisongfox@163.com; 5 Maize Research Institute, Shandong Academy of Agricultural Sciences, Jinan 250100, China; yingboandy@163.com

**Keywords:** deep learning, YOLOv11, maize tassel detection, UAV, lightweight

## Abstract

To tackle the challenges posed by substantial variations in target scale, intricate background interference, and the likelihood of missing small targets in multi-temporal UAV maize tassel imagery, an optimized lightweight detection model derived from YOLOv11 is introduced, named OTB-YOLO. Here, “OTB” is an acronym derived from the initials of the model’s core improved modules: Omni-dimensional dynamic convolution (ODConv), Triplet Attention, and Bi-directional Feature Pyramid Network (BiFPN). This model integrates the PaddlePaddle open-source maize tassel recognition benchmark dataset with the public Multi-Temporal Drone Corn Dataset (MTDC). Traditional convolutional layers are substituted with omni-dimensional dynamic convolution (ODConv) to mitigate computational redundancy. A triplet attention module is incorporated to refine feature extraction within the backbone network, while a bidirectional feature pyramid network (BiFPN) is engineered to enhance accuracy via multi-level feature pyramids and bidirectional information flow. Empirical analysis demonstrates that the enhanced model achieves a precision of 95.6%, recall of 92.1%, and mAP@0.5 of 96.6%, marking improvements of 3.2%, 2.5%, and 3.1%, respectively, over the baseline model. Concurrently, the model’s computational complexity is reduced to 6.0 GFLOPs, rendering it appropriate for deployment on UAV edge computing platforms.

## 1. Introduction

With the continuous growth of the global population and the escalating challenges to agricultural production posed by climate change, food security has become a global issue of common concern to all countries. As one of the world’s three major staple foods, maize occupies a core position in the global food supply system due to its wide adaptability, high yield potential, and diverse applications (including food, feed, and industrial raw materials, among others). It plays an irreplaceable role in ensuring global food security, supporting sustainable agricultural development, and stabilizing economic and social operations [1,2]. The industry of maize seed production stands as the linchpin of maize cultivation, with maize being a monoecious species that undergoes cross-pollination. In its natural state, when the pollen from a feminized tassel alights upon the same ear, self-pollination ensues, yielding inbred seeds that drastically compromise seed purity and weakens the vigor of heterosis—the phenomenon wherein the initial hybrid progeny derived from genetically distinct parents exhibits enhanced attributes in growth, vitality, resilience to stress, yield, and quality—subsequently precipitating a decrement in both the yield and quality of maize, and posing a threat to farmers’ livelihoods. Thus, the meticulous identification and excision of residual tassels post-detasseling of the pistillate parent, are instrumental in preserving seed purity [3]. However, traditional manual detection methods suffer from inherent limitations such as low efficiency and high subjectivity, failing to meet the demands of large-scale modern agricultural production, thus urgently requiring breakthroughs in intelligent detection technologies.

Unmanned Aerial Vehicles (UAVs) equipped with visible light sensors have become an important technical means for crop phenotypic information collection due to their advantages of strong mobility, high cost-effectiveness, and efficient operation [4,5,6,7]. Deep learning technology, with its advantages of high efficiency and non-contact operation, has provided a new approach for automated tassel detection, and relevant research has made positive progress.

In existing studies, scholars have carried out a series of explorations focusing on model lightweighting, accuracy optimization, and scenario adaptability. Yu et al. [8] constructed segmentation models such as PspNet and DeepLab V3 + using RGB images from experimental bases, verifying the universality of U-Net for maize tassel segmentation, but it did not address the issue of complex field scale differences; Liang et al. [9] compared the performance of Faster R-CNN, SSD, and other models in UAV images, and optimized onboard deployment efficiency through SSD_mobilenet; however, the self-built dataset was affected by weather, leading to insufficient model robustness; Zhang et al. [10] replaced the backbone network of YOLOv4 with GhostNet and optimized the activation function, achieving a balance in accuracy, speed, and parameters but did not design dedicated modules for the dynamic morphological characteristics of tassels; Ma et al. [11] improved YOLOv7-tiny by introducing modules such as SPD-Conv and ECA-Net, improving detection accuracy under the premise of lightweighting, yet its adaptability to multi-growth-stage tassel scales remains limited; Du et al. [12] proposed a method combining UAV remote sensing and deep learning to detect maize ears at different stages, with an average precision of 94.5% but the detection performance fluctuates significantly under strong light conditions; Song et al. [13] proposed the SEYOLOX-tiny model, which improved the mean average precision to 95.0% by introducing an attention mechanism but the detection performance for small-sized targets still needs optimization. The above studies all focus on improving the accuracy or efficiency of maize tassel detection, providing important references for this research. However, their improvement directions are mostly focused on single-dimensional optimization (such as backbone network replacement or the introduction of a single attention mechanism), failing to form a systematic solution for complex field scenarios.

Despite the significant progress made by existing methods, tassel detection in multi-temporal UAV images still faces three core challenges that have not been fully addressed by current research: First, tassel morphology evolves dynamically with growth stages, and coupled with differences in UAV flight heights, the target scale span is extremely large. Existing studies mostly rely on single-scale feature extraction networks (e.g., [10,11]), which are difficult to adapt to full-growth-stage scale changes. Second, the complex field environment, including leaf occlusion, uneven illumination, and soil background interference, easily leads to false detection and missed detection. Although some studies have introduced attention mechanisms to enhance feature expression (e.g., [12,13]), they have not solved the problem of information loss in cross-dimensional feature interaction. Third, the computing power at the UAV edge is limited, and the parameter quantity and computational complexity of traditional models restrict real-time detection performance. Existing lightweight schemes (e.g., [9]) mostly achieve efficiency improvement by simplifying network structures, which easily leads to accuracy sacrifice and makes it difficult to balance the dual demands of “accuracy and efficiency”. Therefore, although similar studies have explored intelligent detection methods for maize tassels, there is still an urgent need to develop detection models that are both lightweight and have a high accuracy to meet the actual demand for real-time tasseling stage monitoring in smart agriculture, addressing the challenges of accurate identification of complex germplasm resources and real-time processing through multi-dimensional collaborative optimization.

Based on this, aiming at the limitations of existing methods, this study proposes an OTB-YOLO model integrating multi-module collaborative improvements. Through the enhancement of backbone network features, optimization of neck attention mechanisms, and innovation of multi-scale fusion strategies, it systematically addresses the problems of poor scale adaptability, insufficient robustness in complex environments, and the difficulty in balancing lightweight and accuracy, providing technical support for automated monitoring of tassels in large-scale seed production bases.

## 2. Materials and Methods

### 2.1. Data Preprocessing

This study employs a multi-source heterogeneous dataset, which integrates the open-source maize tassel recognition benchmark dataset from PaddlePaddle with the publicly available Multi-Temporal Drone Corn Dataset (MTDC) [14]. This comprehensive dataset fully covers key growth stages from the tasseling stage to the filling stage, including samples from different geographical regions and varieties, thereby providing the model with abundant phenotypic variation information for learning.

**Figure 1:**
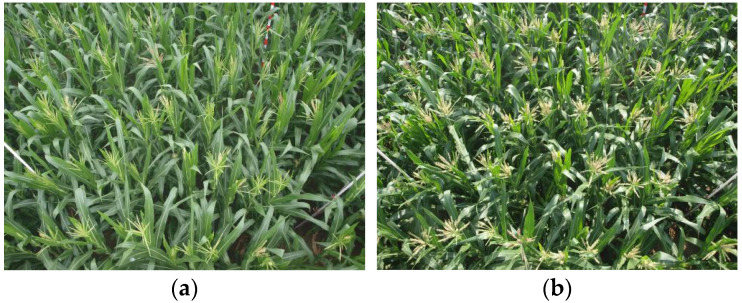
Corn tassel samples at different growth stages. (**a**) Initial tassel emergence, (**b**) Tassel exposure.

To fully leverage the detailed features of high-resolution UAV images (with resolutions of 2736 × 1824 and 3648 × 2736) and ensure the model’s capability to handle large-size inputs, this study directly uses data at the original resolution. This approach sufficiently preserves fine-grained features in the images, such as leaf textures and tassel morphologies, while avoiding target fragmentation or detail loss that may be introduced by block-based operations. The images of the dataset are illustrated in Figure 1.

To enhance the model’s generalization capabilities and ensure experimental consistency, this study employs a two-stage data augmentation strategy. The first stage involves static augmentation applied to the original images during preprocessing. This includes the injection of Gaussian noise (with a standard deviation of σ = 0.05), adjustments to lighting conditions (±30% brightness), and the addition of random salt-and-pepper noise points (with a density ranging from 5 to 10 points per 1000 to 2000 pixels) [15]. Images of low quality, devoid of tassels, or with insufficient plant coverage are excluded from the dataset. The preprocessing stage is particularly attentive to preserving variations in lighting conditions and multi-scale spatial distribution features.

Subsequently, a benchmark dataset is constructed, comprising 2891 high-quality annotated images. During the training phase, the second stage of augmentation is dynamically applied, utilizing mosaic augmentation (four-image stitching) [16]. Professional annotations are meticulously completed using the Labelme tool and then converted into a txt format. The dataset is rigorously divided into training, validation, and test sets, adhering to an 8:1:1 ratio. This meticulous approach provides robust and reliable data support for the maize tassel detection task.

It should be specifically noted that the annotation task of this dataset is strictly limited to a single class—maize tassel. The differences in the samples mentioned in the text, such as “different growth stages (e.g., initial tassel emergence and tassel exposure as shown in Figure 1)”, “different geographical origins”, and “different maize varieties”, only reflect the morphological variations in tassels during natural growth, rather than classifying them into multiple independent classes. All samples are uniformly annotated with the label “maize tassel”, without other crop targets or subclasses.

### 2.2. Evaluation Indicators

In this study, we evaluate the model’s performance using a suite of indicators, including precision (P), recall (R), mean average precision (mAP@0.5), the number of parameters, and computational complexity. Precision quantifies the model’s accuracy in correctly identifying positive samples, effectively indicating the likelihood of false positives where background interference is mistakenly labeled as a target. A higher precision value signifies a lower rate of false positives and denotes better overall performance [17]. Recall measures the model’s ability to capture all actual targets, ensuring that the model’s coverage is comprehensive. The mean average precision at an intersection over union (IoU) threshold of 0.5 (mAP@0.5) provides a holistic assessment of the model’s detection capabilities. Lastly, computational complexity evaluates the intricacy of the model, which is crucial for real-world applications where efficiency is a key factor. Following standard definitions in object detection literature [18,19], the calculations of each indicator are shown in Formulas (1)–(3).

(1) $$P=\frac{TP}{TP+FP}$$

(2) $$R=\frac{TP}{TP+FN}$$

(3) $$mAP@0.5=\frac{AP_{1}+AP_{2}+\ldots +AP_{n}}{n}$$

Here TP (true positives) refers to the number of correctly predicted target samples; FP (false positives) denotes the number of background samples mistakenly predicted as targets; FN (false negatives) represents the number of actual targets missed by the model; and AP denotes the average precision for each category. In this study, since the detection task focuses on a single class (maize tassel), the value of “n” in Formula (3) is 1. That is, mAP@0.5 is equal to the average precision (AP) of this single class, and there is no multi-class classification scenario involved.

## 3. Network Model and Improvements

### 3.1. YOLOv11 Network Architecture

**Figure 2:**
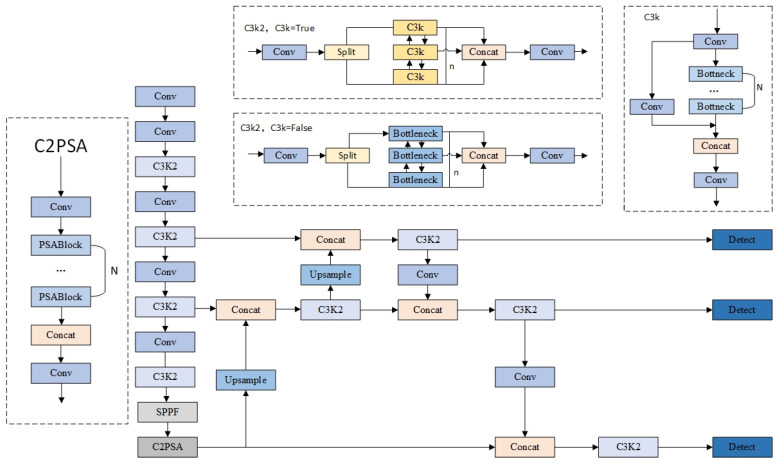
Architectural diagram of the YOLOv11 network. Note: In this architectural diagram, dashed boxes highlight the core innovative modules of YOLOv11 and their configurable logics, including the dual-configuration mode of the C3k2 module denoted as “C3k2 (C3k = True/False)” and the C2PSA mechanism.

YOLOv11, an efficient general-purpose object detection framework developed and open-sourced by Ultralytics [20], features a network architecture comprising three key components: the backbone network, the neck network, and the detection head. Its fundamental structure is illustrated in Figure 2, with core improvements embodied in the design of the following modules:

The backbone network employs the C3K2 module as a replacement for the traditional convolution structure. This module integrates the advantages of standard convolution and group convolution, supporting the selection of C3k units with different convolution kernel sizes or standard bottleneck structures through configuration. It ensures feature extraction efficiency while providing flexible architecture configuration. In the neck network, the C2PSA module is introduced after the SPPF (Spatial Pyramid Pooling Fast) layer. Based on the Pyramid Squeeze Attention mechanism, this module enhances the model’s ability to capture key features through multi-scale feature compression and channel attention weighting. For the detection head, a lightweight prediction layer is constructed using depthwise separable convolution (DWConv). By separating spatial filtering and channel combination operations, it significantly reduces computational complexity while maintaining detection accuracy, with systematic optimization of network depth and width to achieve a balance between computational efficiency and feature expression capabilities.

### 3.2. OTB-YOLO Maize Tassel Detection Model

Leveraging the advantages of the YOLOv11 algorithm, this study proposes a lightweight detection method integrating improved attention mechanisms, namely OTB-YOLO (where “OTB” is derived from the initials of the three core improved modules: ODConv, Triplet Attention, and BiFPN), targeting the specific characteristics of maize tassel detection scenarios (e.g., complex field background interference, diverse tassel morphologies with large scale variations, and the need for lightweight deployment on UAVs). The core innovation of this method lies in achieving a “dual improvement in accuracy and efficiency” through modular improvements: it not only addresses the insufficient capture of tassel features by traditional models but also meets the deployment requirements of UAVs through computational cost optimization. Specifically, the creative structural improvements are as follows:

To tackle the issue that tassel features are easily disturbed in complex field environments, enhanced feature extraction in the backbone is achieved through an independently designed fusion scheme of ODConv (Omni-Dimensional Convolution) and C3K2 modules. The ODConv module is embedded into the C3K2 modules at the last three critical levels of the backbone, strengthening the model’s feature extraction capability while reducing computational costs. For an optimized attention mechanism in the neck, aiming to solve the problem of losing fine morphological information of tassels during feature dimension reduction in traditional attention mechanisms, this study proposes introducing the Triplet Attention mechanism after the feature fusion nodes in the upsampling path of the neck. It enhances the expression of key features (e.g., tassel morphological details) through cross-dimensional interaction, avoiding information loss caused by dimension reduction in traditional attention mechanisms. Regarding the innovative multi-scale feature fusion strategy, considering the significant scale variations in tassel targets captured by UAVs (from small tassels in the seedling stage to large tassels in the mature stage), this study proposes introducing the BiFPN (Bidirectional Feature Pyramid Network) at the feature fusion nodes of both the upsampling and downsampling paths in the neck. It optimizes multi-scale feature fusion through bidirectional cross-layer connections, thereby enhancing the detection robustness of tassel targets at different resolutions.

**Figure 3:**
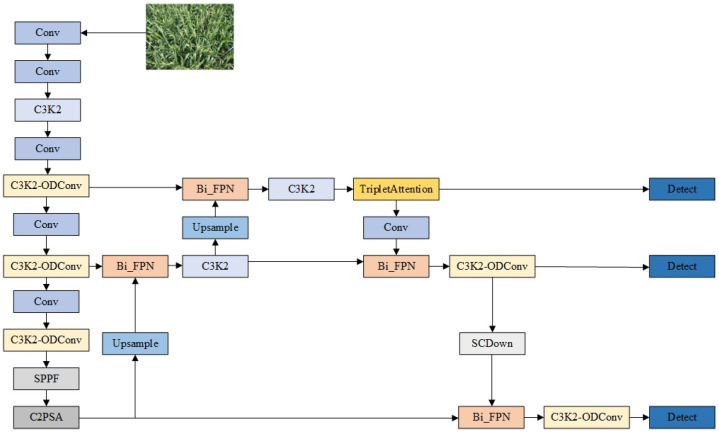
Architectural diagram of the OTB-YOLO network.

The network structure of the improved OTB-YOLO maize tassel detection model is illustrated in Figure 3.

#### 3.2.1. ODConv

**Figure 4:**
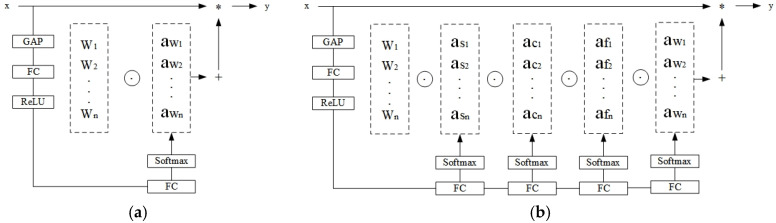
Structural Comparison between Conventional Convolution and ODConv. (**a**) Conventional Convolution, (**b**) ODConv. Here, “x” represents the input feature; “*” denotes the element-wise multiplication operation between the weighted feature and the original feature; “+” stands for the weighted summation operation of multi-weight features; “y” is the output feature after weighted fusion.

The core technical advantage of ODConv [21] lies in its multi-dimensional dynamic attention mechanism. Compared with traditional dynamic convolution, ODConv not only realizes dynamic adjustment of the number of convolution kernels but also innovatively extends to three key dimensions: spatial size, number of input channels, and number of output channels. Figure 4 shows the structural difference between the traditional convolution block and ODConv. This multi-dimensional dynamic characteristic enables ODConv to more finely adapt to the feature changes in maize tassels. Specifically, in dense tassel areas, ODConv can dynamically adjust the spatial size of the convolution kernel to enhance the receptive field, while optimizing the number of input and output channels to highlight key features, thereby effectively avoiding missed and false detections.

Additionally, ODConv uses a parallel strategy to simultaneously learn attention in different dimensions. This parallel architecture not only improves computational efficiency but also ensures the synergistic effect between features in each dimension. In the maize tassel detection task, this strategy allows the network to process multi-dimensional information such as the spatial position, morphological features, and spectral characteristics of tassels in parallel. Through this parallel learning mechanism, ODConv achieves the comprehensive capture of tassel features, while reducing computational redundancy and further optimizing the lightweight characteristics of the model, making it more suitable for deployment on UAVs and other computing-constrained platforms to meet real-time detection requirements.

Therefore, this study adopts the ODConv dynamic convolution technology. By utilizing the multi-dimensional attention mechanism and parallel strategy, it can comprehensively capture the features of maize tassels, thereby improving the accuracy of detection and counting. Through systematic experimental verification, it was finally determined to deploy the C3K2-ODConv module in the last three key feature extraction layers of the backbone and the P4 (medium scale) and P5 (large scale) detection layers of the neck network. This optimization scheme dynamically adjusts convolution kernel parameters and intelligently allocates computing resources, significantly improving the efficiency of feature extraction and downsampling while effectively maintaining the lightweight characteristics of the model, achieving the best balance between detection accuracy and computational efficiency.

#### 3.2.2. Triplet Attention Mechanism

**Figure 5:**
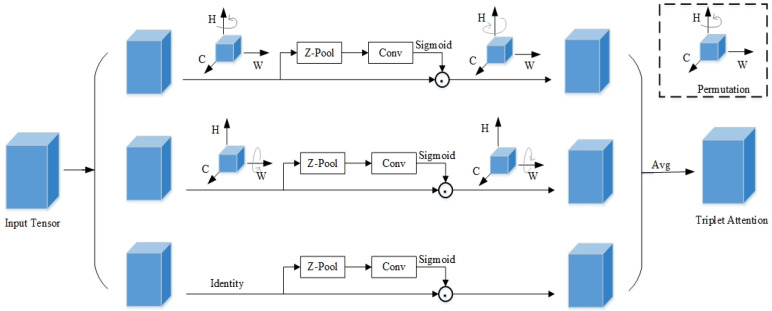
Flowchart of Triplet Attention Implementation.

The Triplet Attention mechanism [22] achieves cross-dimensional feature interaction through a three-branch parallel architecture. Figure 5 shows the specific implementation flowchart of Triplet Attention, demonstrating how the three branches process the input tensor and finally synthesize triplet attention. It mainly includes four key steps: first, the input features are, respectively, converted into interactive representations of different dimensions through tensor permutation transformation, including channel-height, height-width, and width-channel interactions; subsequently, each branch compresses global features through Z-pooling, captures local details in combination with k × k convolution, and generates attention weights using the Sigmoid function; then, the generated attention weights are subjected to Hadamard product with the transformed features, and finally, the original dimensions are restored through inverse transformation and fused with weights to obtain the triplet attention output.

This structure establishes the interaction relationship between channels and spatial dimensions through different tensor rotation and permutation operations, thereby comprehensively extracting the morphological features of the detection target. In addition, the mechanism realizes lightweight design by sharing convolution kernel parameters and feature reuse between branches, reducing the model’s computational burden. In the maize tassel detection task, this study deploys the Triplet Attention module in the small target detection layer. Through the unique three-branch structure, it captures the cross-dimensional interaction information of the input data and efficiently calculates the attention weights, strengthening the network’s perception ability to target features in the detection task and enhancing the model’s ability to capture multi-scale and multi-angle features of maize tassels. At the same time, through its lightweight design, while reducing the computational burden, it constructs the interdependence between input channels and spatial positions, enhances the model’s ability to capture multi-scale and multi-angle features of maize tassels, focuses precisely on the key features of tassels, and avoids missed detections caused by complex backgrounds or lighting changes. It is particularly suitable for complex scenarios such as missed detection of small tassels and uneven distribution in UAV images.

#### 3.2.3. BiFPN Bidirectional Feature Pyramid Network

**Figure 6:**
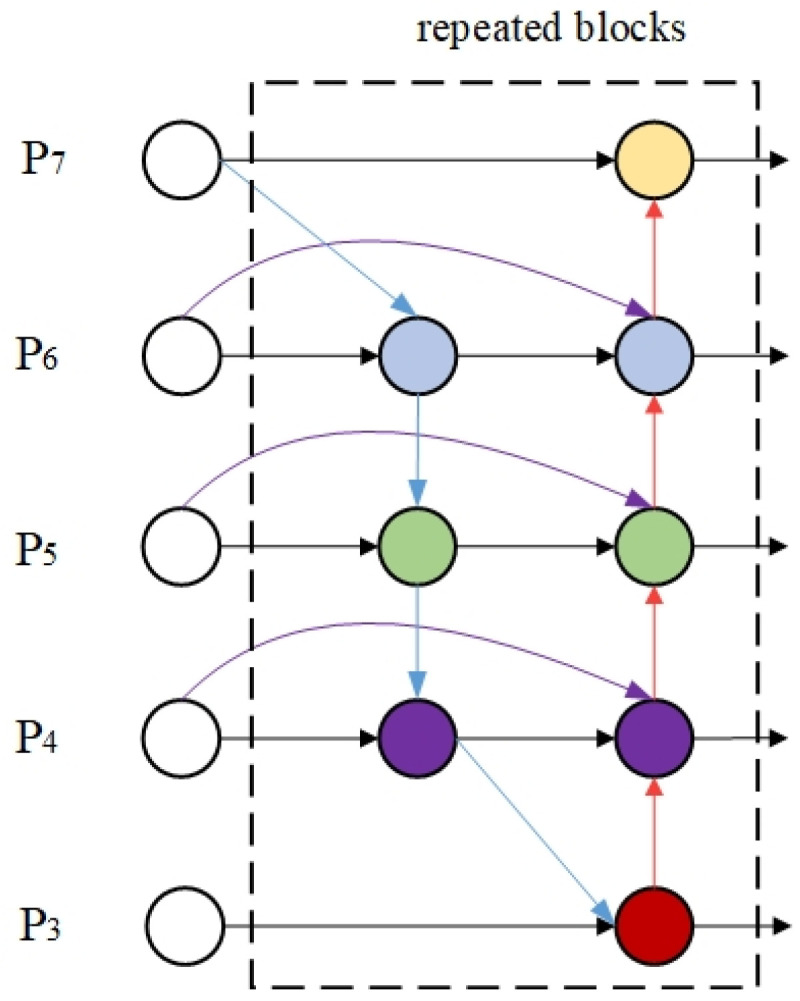
Architecture of BiFPN.

The BiFPN (Bidirectional Feature Pyramid Network) [23] realizes efficient feature transfer and information interaction through a multi-scale feature fusion mechanism, and its network structure is shown in Figure 6. This structure introduces bidirectional cross-scale connections on the basis of the traditional feature pyramid. First, it performs top-down feature transfer on the input features to fuse high-level semantic information; subsequently, it integrates low-level detail features through the bottom-up path; finally, it realizes dynamic fusion of multi-scale features through learnable feature weights.

Aiming at the problem of feature extraction for targets of different scales at different growth stages in maize tassel detection, this study introduces the BiFPN module into the neck network to improve the model’s ability to fuse tassel detail and global features through constructing a bidirectional feature fusion mechanism. BiFPN breaks through the limitation of unidirectional feature transfer and realizes efficient interaction of features at different scales by constructing bidirectional connection paths from top to bottom and from bottom to top. In the maize tassel detection scenario, this mechanism allows the detailed edge information of tassels captured by shallow networks (such as branch texture and tassel axis morphology) to be fully fused with the semantic features extracted by deep networks (such as overall contour and spatial distribution), effectively solving the problems of missed detection of small tassels and false detection of large tassels. At the same time, BiFPN reduces computational complexity by deleting redundant single-input nodes, adding direct connection edges between same-level nodes, and modularly reusing bidirectional paths, strengthening the transfer efficiency of cross-scale features.

## 4. Experiments and Results

### 4.1. Experimental Operation Environment

The hardware device used in this study is a computer equipped with an Intel (R) Core (TM) i7-14700KF processor and an NVIDIA GeForce RTX 4060Ti graphics processing unit (8 GB video memory). The core parameters are presented in Table 1. The operating system is Windows 11, the deep learning framework is PyTorch 2.5.0+cu124, and the programming language is Python 3.10. All comparison algorithms are executed in the same environment.

The training parameters are set as follows: the batch size is set to 8, which not only improves the model iteration efficiency but also avoids excessive memory occupation to ensure the smooth progress of training; the number of training iterations (epochs) is set to 100, which allows the model to fully learn data patterns and effectively prevents overfitting, ensuring good generalization ability, and makes the comparison between models more obvious; and in the actual training stage, the AdamW optimizer is used, with an initial learning rate (lr) of 0.01 and a momentum value of 0.9, which helps the model converge quickly and stably to find optimal parameters.

### 4.2. Ablation Experiments

To evaluate the impact of each improved module on model performance, this study uses YOLOv11n as the baseline model for ablation test verification. The specific test results are shown in Table 2. The results in this table are all obtained from inference on the test dataset described at the end of Section 2.1.

**Figure 7:**
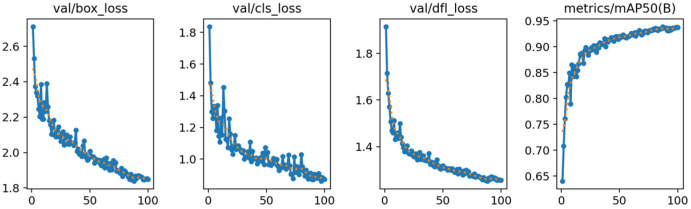
Performance metrics curve of OTB-YOLO. Here, “val” refers to the validation set. “val/box_loss” is the error in predicting tassel bounding boxes, with lower values indicating more accurate localization; ”val/cls_loss” is the classification error between tassels and non-tassel elements (e.g., leaves), with a downward trend indicating better classification performance; “val/dfl_loss” is the error in judging tassel presence, with lower values indicating fewer false positives and missed detections; ”metrics/mAP50(B)” is the average precision at 50% Intersection over Union (IoU), where “(B)” denotes “Bounding box”; higher values (approaching 1) indicate better detection quality.

It can be seen from Table 2 that compared with YOLOv11n, each improved model has improvements in precision, recall, and mAP@0.5, and with the superposition of modules, the performance is further improved. Finally, the OTB-YOLO model achieves a precision of 95.6%, a recall of 92.1%, and an mAP@0.5 of 96.6%, which are 3.2%, 2.5%, and 3.1% higher than the baseline model, respectively, demonstrating the best detection effect. In terms of the number of parameters and computational complexity, although some improved models have increased, the range is controllable. With the support of the BiFPN and ODConv modules, the model is more lightweight. Finally, the computational complexity of OTB-YOLO is controlled at 6.0 GFLOPs, which is a 4.8% decrease compared with the original model. While improving performance, it takes into account computational resource consumption, proving that the combination of these modules can effectively optimize the model’s ability to detect maize tassels. In the model performance analysis section, the training process performance of OTB-YOLO was evaluated. To clearly present the model’s performance on independent data, Figure 7 selects key metrics from the validation set and retains four subfigures, intuitively illustrating the core performance changes in the model during the testing phase. From the changes in performance metric data, it can be observed that the model has reached a converged state. Specifically, this is characterized by the stabilization of validation set losses (val/box_loss, val/cls_loss, val/dfl_loss), which no longer decrease significantly with training iterations. The stabilization of loss metrics indicates that the model has sufficiently learned the data patterns, with its ability to control errors in tassel localization, classification, and presence judgment reaching a stable level. Meanwhile, the core evaluation metric, metrics/mAP50(B), shows slowed growth and approaches its peak value, a peak that directly verifies that the model’s detection accuracy has reached an optimal state. These phenomena indicate that the model’s prediction errors on the validation set have dropped to a low level, and the detection accuracy has stabilized.

These results confirm that the integration of ODConv, Triplet Attention, and BiFPN is not only additive but synergistic: ODConv reduces redundant computations to lay a lightweight foundation, Triplet Attention enhances feature discrimination to reduce missed detections, and BiFPN optimizes multi-scale fusion to improve overall precision. Their combined effect enables OTB-YOLO to outperform the baseline model in both accuracy and efficiency, which aligns with the design philosophy reflected in its naming.

### 4.3. Performance Comparison of Different Object Detection Algorithms

To evaluate the superiority of the OTB-YOLO model proposed in this study compared with other mainstream convolutional neural networks in the maize tassel detection task, the algorithm in this study was compared with Faster-RCNN, SSD, RetinaNet, YOLOv5s, and YOLOv8n algorithms under the same conditions (including consistent input image size, training epochs, and hardware environment). In addition to key metrics such as precision, recall, mAP@0.5, parameters, and computational complexity, a new column, “Inference Time/ms”, has been added to reflect the actual execution efficiency of each model in practical applications. The test results are shown in Table 3.

Specifically, as a typical two-stage detection algorithm, Faster-RCNN [24] achieves a precision of 92.5% and an mAP@0.5 of 89.7%, demonstrating high detection accuracy, but its computational cost is relatively high and its inference time is 178 ms, reflecting its relatively slow detection speed. As a representative of early single-stage algorithms, SSD [25] has the advantages of few parameters (2.2 M) and low computational complexity (11.4GFLOPs), and its inference time is 48.1 ms, showing a relatively fast detection speed, but its detection accuracy is relatively limited (mAP@0.5 = 79.2%). RetinaNet [26] improves the problem of category imbalance to a certain extent by introducing the Focal Loss mechanism (mAP@0.5 = 88%), but its complex network structure leads to a parameter count as high as 38.6 M and a computational complexity of 206GFLOPs, and its inference time is 244.2 ms, with limited performance improvement in complex field scenarios and relatively slow speed. Although YOLOv8 is improved compared with YOLOv5, and the inference time of both is relatively short, its detection accuracy is still lower than that of YOLOv11. In contrast, the OTB-YOLO model proposed in this study performs the best, not only significantly leading in detection accuracy but also maintaining excellent lightweight characteristics, fully verifying the superiority and practicality of the model in the maize tassel detection task.

### 4.4. Visual Comparison and Verification

**Figure 8:**
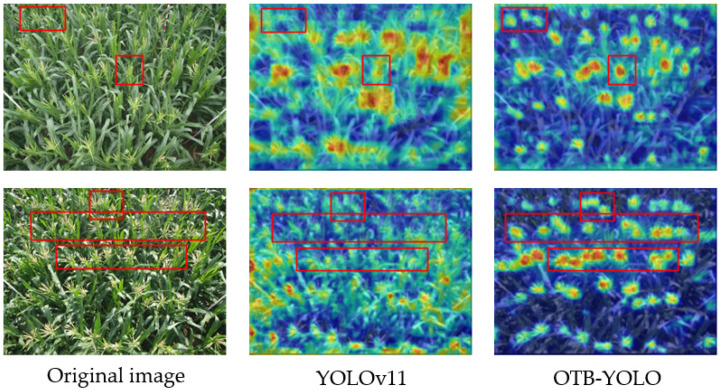
Comparison of Grad-CAM Heatmaps. Red boxes highlight regions with significant attention differences, enabling direct visual comparison.

To deeply analyze the model performance, this study uses the Grad-CAM [27] method for visualization analysis of the detection process, as shown in Figure 8. The heatmap results show that the OTB-YOLO model exhibits more precise attention distribution characteristics which are as follows: (1) highly concentrated activation responses in key tassel regions (such as tassel axes and branch structures); (2) significant suppression of background interferences (such as leaves and soil). In contrast, the activation regions of the baseline model show obvious attention dispersion, and some non-target regions (such as adjacent leaves) have wrong activations. This indicates that the OTB-YOLO model shows better performance in understanding the task. Notably, the red-framed areas in the original images highlight representative tassel targets (like small-sized and densely distributed ones). In these red-framed regions, YOLOv11 has weak feature responses (with dim heatmap colors and blurred boundaries), prone to missed or false detections. However, OTB-YOLO shows clear and accurate responses in the corresponding positions, vividly demonstrating its advantages in detecting such challenging targets.

## 5. Discussion

To address issues such as feature interference, detail loss, and poor scale adaptability in maize tassel detection from UAV images, this study proposes the OTB-YOLO model based on improved YOLOv11. Three core optimizations are implemented to enhance performance: replacing traditional convolution with omni-dimensional dynamic convolution (ODConv) to reduce computational redundancy; introducing a Triplet Attention module to strengthen the feature extraction capability of the backbone network; and designing a bidirectional feature pyramid network (BiFPN) to optimize multi-scale feature fusion.

Ablation experiments validate the synergistic effectiveness of the improved modules: the model achieves a precision of 95.6% (+3.2%), a recall of 92.1% (+2.5%), and an mAP@0.5 of 96.6% (+3.1%), while maintaining lightweight characteristics with 3.4 M parameters and 6.0 GFLOPs. Specifically, ODConv dynamically adapts to morphological changes in maize tassels, compensating for the lack of dedicated dynamic feature modules in Zhang et al.’s [10] model; the Triplet Attention module enhances the expression of key features through cross-dimensional interaction, effectively alleviating the poor robustness of Liang et al. ’s [9] model caused by environmental interference; BiFPN improves the efficiency of multi-scale fusion via bidirectional cross-layer connections, breaking through the limitation of Ma et al. ’s [11] model in scale adaptation for maize tassels across multiple growth stages. Together, these modules achieve a balanced optimization of “precision-efficiency” in complex field scenarios.

Comparative experiments with mainstream models such as Faster-RCNN, SSD, RetinaNet, YOLOv5, and YOLOv8 further confirm the superiority of OTB-YOLO. Its detection accuracy (precision: 95.6%, recall: 92.1%, mAP@0.5: 96.6%) significantly outperforms the comparison models, and its lightweight characteristics (3.4 M parameters, 6.0 GFLOPs) are superior to most similar algorithms. Compared with Song et al. ’s [13] SEYOLOX-tiny model (mAP@0.5 of 95.0%), OTB-YOLO not only achieves a 1.6 percentage point improvement in accuracy but also optimizes the detection performance for small-sized tassels through the enhanced extraction of small-scale features by BiFPN, thereby addressing the drawback of its high missed detection rate for small targets. Grad-CAM visualization analysis intuitively shows that the model exhibits more precise attention distribution on key regions of maize tassels, such as tassel axes and branches, providing a mechanistic explanation for its performance advantages.

## 6. Conclusions

This study successfully develops the OTB-YOLO model by improving YOLOv11, effectively addressing key challenges in maize tassel detection from UAV images, including feature interference, detail loss, and poor scale adaptability. The integration of three core optimizations—omni-dimensional dynamic convolution (ODConv), Triplet Attention module, and bidirectional feature pyramid network (BiFPN)—synergistically enhances detection performance while maintaining lightweight properties (3.4 M parameters, 6.0 GFLOPs). Experimental results validate that OTB-YOLO achieves superior accuracy metrics (precision: 95.6%, recall: 92.1%, mAP@0.5: 96.6%) compared to existing models and effectively mitigates limitations of prior studies, such as poor dynamic feature adaptation and high small-target missed detection rates.

Future research will focus on exploring multi-modal data fusion strategies (e.g., thermal infrared) to further improve the model’s detection robustness in complex environments. Ultimately, the goal is to construct an intelligent monitoring platform covering the entire growth cycle of maize, providing reliable technical support for precision agricultural field management.

## Figures and Tables

**Table 1:** Hardware Parameters.

Hardware Components	Model/Specification	Core Parameters
Central Processing Unit (CPU)	Intel (R) Core (TM) i7-14700KF	Architecture: Raptor Lake-S Efficiency cores 2.5 GHz Logical Processors: 28 Cores: 20 Base Speed: 3.4 GHz
Graphics Processing Unit (GPU)	NVIDIA GeForce RTX 4060Ti	Architecture: Ada Lovelace (AD106 core) Number of CUDA Cores: 4352 Memory: 8 GB GDDR6 (128-bit width) Core Frequency: Base 2310 MHz, Boost 2535 MHz Memory Bandwidth: 224 GB/s
Memory	CT16G56C46U5.C8B2	Capacity: 32 GB (dual-channel, 16 GB × 2) Speed: 5600 MT/s Frequency: 2800 MHz

**Table 2:** Results of Ablation Experiments.

Model	Precision/%	Recall/%	mAP@0.5/%	Parameters/M	Computational Complexity/GFLOPs
YOLOv11n	92.4	89.6	93.5	2.6	6.3
YOLOv11n+ODConv	93.3	90.3	94.3	3.2	6.1
YOLOv11n+Triplet Attention	94.1	90.7	94.8	2.7	6.6
YOLOv11n+ODConv+Triplet Attention	94.6	91.7	95.7	3.2	6.4
YOLOv11n+Triplet Attention+BiFPN	94.8	91.3	95.8	2.6	6.3
OTB-YOLO	95.6	92.1	96.6	3.4	6.0

**Table 3:** Results of Comparative Experiments.

Model	Precision/%	Recall/%	mAP@0.5/%	Parameters/M	Computational Complexity/GFLOPs	Inference Time/ms
Faster-RCNN	92.5	89.4	89.7	14.3	33.2	178
SSD	80.2	75.1	79.2	2.2	11.4	48.1
RetinaNet	92	86.9	88	38.6	206	244.2
YOLOv5s	89.6	91.2	88.4	5.8	2.2	31
YOLOv8n	89.8	91.8	93.7	8.1	3.0	25.7
OTB-YOLO	95.6	92.1	96.6	3.4	6.0	61.2

## Data Availability

The raw data supporting the conclusions of this article will be made available by the authors on request.
